# Efficacy of plant breeding using genomic information

**DOI:** 10.1002/tpg2.20346

**Published:** 2023-05-04

**Authors:** Osval A. Montesinos‐López, Alison R. Bentley, Carolina Saint Pierre, Leonardo Crespo‐Herrera, Leonardo Rebollar‐Ruellas, Patricia Edwigis Valladares‐Celis, Morten Lillemo, Abelardo Montesinos‐López, José Crossa

**Affiliations:** ^1^ Facultad de Telemática Universidad de Colima Colima México; ^2^ International Maize and Wheat Improvement Center (CIMMYT) Estado de México México; ^3^ Independent consultant Colima México; ^4^ Bachillerato 22 Universidad de Colima Cuauhtémoc Colima México; ^5^ Department of Plant Sciences Norwegian University of Life Sciences Ås Norway; ^6^ Centro Universitario de Ciencias Exactas e Ingenierías (CUCEI) Universidad de Guadalajara Guadalajara Jalisco México; ^7^ Colegio de Postgraduados Montecillos Estado de México México

## Abstract

Genomic selection (GS) proposed by Meuwissen et al. more than 20 years ago, is revolutionizing plant and animal breeding. Although GS has been widely accepted and applied to plant and animal breeding, there are many factors affecting its efficacy. We studied 14 real datasets to respond to the practical question of whether the accuracy of genomic prediction increases when considering genomic as compared with not using genomic. We found across traits, environments, datasets, and metrics, that the average gain in prediction accuracy when genomic information is considered was 26.31%, while only in terms of Pearson's correlation the gain was of 46.1%, while only in terms of normalized root mean squared error the gain was of 6.6%. If the quality of the makers and relatedness of the individuals increase, major gains in prediction accuracy can be obtained, but if these two factors decrease, a lower increase is possible. Finally, our findings reinforce genomic is vital for improving the prediction accuracy and, therefore, the realized genetic gain in genomic assisted plant breeding programs.

## Core Ideas

- Although genomic selection (GS) has been widely accepted and applied to plant, there are many factors affecting its efficacy.
- Genomic prediction models are built based on the relationship between genetic markers and the traits of interest using a training population.
- If the quality of the makers and relatedness of the individuals increase, major gains in prediction accuracy can be obtained, but if these two factors decrease, a lower increase is possible.

**Abbreviations**
BLUP: best linear unbiased predictor
Cor: correlation
EHT: early heat
EYT: elite yield trial
GBLUP: genomic best linear unbiased predictor
GS: genomic selection
GY: grain yield
IR: irrigation
LHT: late heat
NRMSE: normalized root mean squared error
RE: relative efficiency
YT: yield trial

## 1 INTRODUCTION

Genomic selection (GS) is a predictive methodology proposed by Meuwissen et al. (2001), which has revolutionized plant breeding because breeders can select potential candidates before they are planted in the field. Researchers train a statistical machine learning model with recorded phenotypic and genotypic information of a reference population (also called training population), then engage it to make predictions regarding individuals for which only genotypic information is available (Desta & Ortiz, 2014).

This predictive methodology is called genomic selection because it requires high‐density markers across the whole genome and depends on the assumption that at least one genetic marker will be in linkage disequilibrium with a causative quantitative trait locus for the trait of interest (Meuwissen et al., 2001). Successful implementation of GS depends on many factors, including: (a) the heritability of the trait of interest, (b) relationships of the individuals in the training and testing set (Habier et al., al. 2007), (c) the size of the training and testing sets, (d) the goal of prediction, for example, tested lines in tested environments, untested lines in tested environments, tested lines in untested environments or untested lines in untested environments, (e) the statistical machine learning method implemented for obtaining the predictions, (f) marker density, and (g) population structure (Frouin et al., 2019).

Advantages of GS over traditional (phenotypic) selection include the following: (a) the possibility of selecting individuals before they are planted, (b) the potential for selecting superior genotypes with higher precision, (c) reduced costs, in part by saving resources required for extensive phenotyping, (d) less time needed for variety development by reducing the cycle length, (e) the ability to substantially increase the selection intensity, thus providing scenarios for capturing greater gain per unit time, (f) the ability to select difficult to measure traits, and (g) increased selection process accuracy.

Because GS promises more precise selection and shorter generation intervals to improve breeding efficiency (Ortiz, 2015), it is being implemented in many crops such as wheat, maize, cassava, rice, chickpea, and groundnut (Crossa et al., 2017; Huang et al., 2019; Roorkiwal et al., 2016; Wolfe et al., 2017). Nevertheless, practical application of GS is complex because successful implementation is determined by the predictive accuracy of the statistical machine learning methods employed.

Our goal in this research was to exhibit the importance of the genomic markers information to improve the prediction accuracy in plant breeding trials. Using 14 real datasets (some with several traits), we compared the prediction performance of not using (Method A) and using (Method B) genomic information in the prediction models. For the benchmark, we used the Bayesian best linear unbiased predictor model (BLUP) with the effects of environment, genotypes, and genotypes × environment interaction.

**Core Ideas**

- Although genomic selection (GS) has been widely accepted and applied to plant, there are many factors affecting its efficacy.
- Genomic prediction models are built based on the relationship between genetic markers and the traits of interest using a training population.
- If the quality of the makers and relatedness of the individuals increase, major gains in prediction accuracy can be obtained, but if these two factors decrease, a lower increase is possible.

## 2 MATERIALS AND METHODS

### 2.1 Bayesian BLUP model (Model A)

The model that does not include genomic information is as follows:

(1) $$\;Y_{ij}\;=\;\;\mu \;+\;E_{i}\;+\;L_{j}\;+\;LE_{ij}\;+\;\varepsilon_{ij},\;$$

where $Y_{ij}$ represents the response variable measured in the *j*th line and *i*th environment, and μ is a general intercept. The environments ($E_{i}$) are considered random variables with identically and independent normal distribution (iid) $N(0,I\sigma_{E}^{2})$ with mean 0 and variance $\sigma_{E}^{2}$, the cultivars ($L_{j}$) are iid random variables with normal distribution $N(0,I\sigma_{L}^{2})$ with mean 0 and variance $\sigma_{L}^{,\;2}$, the cultivar × environment interaction $\left(LE_{ij}\right)$ are iid random variables with normal distribution $N(0,I\sigma_{LE}^{2})$ with mean 0 and variance $\sigma_{LE}^{2}$, and $\varepsilon_{ij}$ are random error components in the model assumed to be iid with mean 0 and variance$\;\sigma_{e}^{2}$.

### 2.2 Bayesian genomic best linear unbiased predictor (GBLUP) model (Model B)

The implemented single‐trait statistical genomic model was as follows:

(2) $$\;Y_{ij}\;=\;\;\mu \;+\;E_{i}\;+\;g_{j}\;+\;gE_{ij}\;+\;\varepsilon_{ij}\;,$$

where $Y_{ij},$μ, $E_{i}$ and $\varepsilon_{ij}$ are defined as in equation (1), but $g_{j},$ j=1,…,J, are the random effects of genotypes, $gE_{ij}$ are the random effects of genotype × environment interaction. Furthermore, it is assumed that $g\;={\left(g_{1},\text{…},g_{J}\right)}^{T}\;∼N_{J}\left(0,\sigma_{g}^{2}G\right)$, $\mathrm{gE}\;={\left(gE_{11},\text{…},gE_{1J},\text{…},gE_{IJ}\right)}^{T}\;∼N_{IJ}\left[0,\sigma_{gL}^{2}\left(Z_{E}Z_{E}^{T}⊙Z_{g}GZ_{g}^{T}\right)\right]$, where **G** is the genomic relationship matrix (linear kernel) computed as proposed by VanRaden (2008) and *T* denotes matrix transpose. The implementation of both models was carried out in the R statistical software (R Core Team, 2022) using the Bayesian generalized linear regression library of Pérez and de los Campos (2014).

#### 2.2.1 Datasets

##### 2.2.1.1 Datasets wheat EYT_1, EYT_2, and EYT_3

These datasets are elite yield trial (EYT) cycles 2013—2014, 2014–2015, and 2015–2016 (EYT_1, EYT_2, and EYT_3, respectively) from the Global Wheat Program of the International Maize and Wheat Improvement Center (CIMMYT) and belong to elite yield trials (EYT) established in four different cropping seasons with four or five environments in each. The lines involved in this study correspond to years 2013–2014, 2014–2015, and 2015–2016. The EYT_1, EYT_2, and EYT_3, contain 776, 775, and 964 lines, respectively. The experimental design was alpha lattice, and the lines were sown in 39 trials, each covering 28 lines and two checks in six blocks with three replications. In these datasets, several traits were available for some environments and lines. We included four traits measured for each line in each environment: days to heading (number of days from germination to 50% spike emergence), days to maturity (number of days from germination to 50% physiological maturity or the loss of the green color in 50% of the spikes), plant height, and grain yield (GY). For full details of the experimental design and how the best linear unbiased estimates were computed, see Juliana et al. (2018).

Wheat lines from datasets EYT_2, and EYT_3 were evaluated in five environments, and wheat lines from dataset EYT_1 was evaluated in four environments. For EYT_1, the environments were bed planting with five irrigations (Bed5IR), early heat (EHT), flat planting and five irrigations (Flat5IR), and late heat (LHT). For EYT_2, the environments were bed planting with two irrigations (Bed2IR), Bed5IR, EHT, Flat5IR, and LHT, while for dataset EYT_3, the environments evaluated were Bed2IR, Bed5IR, Flat5IR, flat planting with drip irrigation (FlatDrip) and LHT.

Genome‐wide markers for the 2515 (776 + 775 + 964) lines in the two datasets were obtained using genotyping‐by‐sequencing (GBS) (Elshire et al., 2011; Poland et al., 2012) at Kansas State University using an Illumina HiSeq2500. After filtering, 2038 markers were obtained from an initial set of 34,900 markers. The imputation of missing markers data was carried out using LinkImpute (Money et al., 2015) and implemented in trait analysis by association evolution and linkage (TASSEL) (Bradbury et al., 2007), version 5. Lines with more than 50% missing data were removed, and a total of 2515 lines across the three datasets were used in this study.

##### 2.2.1.2 Dataset Groundnut

The phenotypic dataset reported by Pandey et al. (2020) contains information on the phenotypic performance for various traits in four environments. In our study, we assessed predictions using the trait seed yield per plant for 318 lines in four environments denoted as Environment1 (ENV1): Aliyarnagar_Rainy 2015; Environment2 (ENV2): Jalgoan_Rainy 2015; Environment3 (ENV3): ICRISAT_Rainy 2015; and Environment4 (ENV4): ICRISAT Post‐Rainy 2015.

The dataset is balanced, with a total of 1272 assessments in each line included once in each environment. Marker data were available for all lines and 8268 single nucleotide polymorphism (SNP) markers remained after quality control (each marker was coded with 0, 1, or 2).

##### 2.2.1.3 Dataset Maize

This dataset was included in Souza et al. (2017); and is 722 (with 722 ×4= 2888 observations) maize hybrids obtained by crossing 49 inbred lines. The hybrids were evaluated in four environments (Env1–Env4) in Piracicaba and Anhumas, São Paulo, Brazil, in 2016 where only GY was the trait included in this study. The hybrids were evaluated using an augmented block design, with two commercial hybrids as checks to correct for microenvironmental variation. At each site, two levels of nitrogen (N) fertilization were used. The experiment conducted under ideal N conditions received 100 kg ha^−1^ of N (30 kg ha^−1^ at sowing and 70 kg ha^−1^in a coverage application) at the V8 plant stage, while the experiment with low N received 30 kg ha^−1^ of N at sowing. The parent lines were genotyped with an Affymetrix Axiom Maize Genotyping Array of 616 K SNPs. Markers with minor allele frequency (MAF) of 0.05 were removed. After quality control, 54,113 SNPs were available.

##### 2.2.1.4 Dataset Disease

This dataset contains 438 wheat lines for which three diseases were recorded: *Pyrenophora tritici‐repentis* (PTR) which causes infections known as helminthosporiosis, tan spot, yellow leaf spot, or yellow leaf blotch. Second, *Parastagonospora nodorum*, a major fungal pathogen of wheat fungal taxon which includes several plant pathogens affecting the leaves and other parts of the plant. Third, *Bipolaris sorokiniana*, which is economically important as the cause of seedling diseases, common root rot and spot blotch of barley, wheat, and several other crops. The 438 wheat lines were evaluated in the greenhouse for several replicates. The replicates were considered as different environments (Env1, Env2, Env3, Env4, Env5, and Env6). The total number of observations were 438 ×6 = 2628 observations for which the three traits were measured.

DNA samples were extracted from each line, following the manufacturer's protocol. DNA samples were genotyped using 67,436 SNPs. For a given marker, the genotype for the *i*th line was coded as the number of copies of a designated marker‐specific allele carried by the *i*th line (absence = zero and presence = one). SNP markers with unexpected heterozygous genotypes were recoded as either AA or BB. We included markers that had less than 15% missing values. Then, we imputed the markers using observed allelic frequencies. We also removed markers with MAF < 0.05. After quality control and imputation, a total of 11,617 SNPs were available for analysis.

##### 2.2.1.5 Datasets wheat YT_1–6

Spring wheat lines selected for GY (only trait included in this study) analyses from CIMMYT first year yield trials (YT) were used as the training population to predict the quality of lines selected from EYT for GY analyses in a second year. The analyses were conducted for GY using six sets of data, as reported below:

– YT_1 (2013–2014/2014–2015), 1301 lines from the 2013–2014 YT and 472 lines from the 2014–2015 EYT trial. In this dataset, only the GY trait was used.
– YT_2 (2014–2015/2015–2016), 1337 lines from the 2014–2015 YT and 596 lines from the 2015–2016 EYT trial.
– YT_3 (2015–2016/2016–2017), 1161 lines from the 2015–2016 YT and 556 lines from the 2016–2017 EYT trial.
– YT_4 (2016–2017/2017–2018), 1372 lines from the 2016–2017 YT and 567 lines from the 2017–2018 EYT trial.
– YT_5 (2017–2018/2018–2019), 1386 lines from the 2017–2018 YT and 509 lines from the 2018–2019 EYT trial.
– YT_6 (2018–2019/2019–2020), 1276 lines from the 2018–2019 YT and 124 lines from the 2019–2020 EYT trial.

More details of these datasets can be found in Ibba et al. (2020).

All the lines were genotyped using GBS (Poland et al., 2012). The TASSEL v.5 GBS pipeline was used to call marker polymorphisms, and a MAF of 0.01 was used for SNP discovery. The resulting 6,075,743 unique tags were aligned to the wheat genome reference sequence (RefSeq v.1.0) with an alignment rate of 63.98%. After filtering for SNPs with homozygosity > 80%, *p*‐value for Fisher's exact test < 0.001 and *χ* ^2^ value lower than the critical value of 9.2, we obtained 78,606 GBS markers that passed at least one of those filters. These markers were further filtered for less than 50% missing data, greater than a 0.05 MAF and less than 5% heterozygosity in all the datasets. Markers with missing data were imputed using the “expectation‐maximization” algorithm in the “R” package rrBLUP (Endelman, 2011).

##### 2.2.1.6 Dataset Indica

The rice dataset was reported by Monteverde et al. (2019) and contains information on the phenotypic performance of four traits (GY; PHR, percentage of head rice recovery; GC, percentage of chalky grain; and PH, plant height) in three environments (years 2010, 2011, and 2012). Three hundred twenty seven lines were evaluated in each year and 54 environmental covariates. This dataset is balanced, with 981 assessments with each line included in each environment. Marker data were available for all lines and 16,383 SNP markers remained after quality control (each marker was coded with 0, 1, or 2).

##### 2.2.1.7 Dataset Japonica

This rice dataset belongs to the tropical Japonica population, was reported by Monteverde et al. (2019) and contains phenotypic information on the same four traits as for the Indica population (GY, PHR, GC, and PH), but evaluated in five environments (years 2009, 2010, 2011, 2012, and 2013). For 2009, 2010, 2011, 2012, and 2013, 93, 292, 316, 316, and 134 lines were evaluated, respectively. Also, the same 54 environmental covariates measured in the Indica dataset (Dataset 13), were also not used. Since this dataset is not balanced, 1051 assessments were evaluated in the five environments. Marker data were available for 320 lines and 16,383 SNP markers remained after quality control (each marker was coded with 0, 1, and 2).

#### 2.2.2 Data availability

The phenotypic and genomic information for the non‐wheat datasets (datasets from Groundnuts, Maize, Disease, Indica, and Japonica) employed on this study can be downloaded from the link: https://github.com/osval78/PLS‐data. The two wheat datasets, EYT_1–3 and YT_1–6 can be found in Juliana et al. (2018) and Ibba et al. (2020), respectively.

#### 2.2.3 Metrics for evaluation of prediction accuracy

In each of the 14 datasets, the seven outer fold cross‐validation was implemented (Montesinos‐López et al., 2022). For this reason, 7 − 1 folds were assigned to the training set and the remaining were assigned to the testing set, until each of the seven folds were used as testing set once. Next, the average of the seven folds was reported as prediction performance using Pearson´s correlation (Cor) and the normalized root mean square error (NRMSE), computed as $\mathrm{NRMSE}\;\;=\;\frac{\mathrm{RMSE}}{\bar{y}}\;$, where RMSE = $\sqrt{\frac{1}{T}\left(\sum_{i=1}^{T}{\left(y_{i}-\hat{f}\left(x_{i}\right)\right)}^{2}\right)}\;$, with $y_{i}$ denoting the observed value *i*, while $\hat{f}\left(x_{i}\right)$ represents the predicted value for observation *i* and $\bar{y}$ is the mean value of the observed values. To compare the two methods using (Method B) and not using (Method A) genomic information was computed the relative efficiencies in terms of NRMSE as follows:

$${\mathrm{RE}}_{\mathrm{NRMSE}}\;=\;\frac{{\mathrm{NRMSE}}_{\mathrm{Method}\;A}}{{\mathrm{NRMSE}}_{\mathrm{Method}\;B}}\;,$$

where RMSE_Method A_ and NRMSE_Method B_ denotes the NRMSE of Methods A and B, respectively. While in terms of Cor, the RE was computed as follows:

$${\mathrm{RE}}_{\mathrm{Cor}}\;=\;\frac{{\mathrm{Cor}}_{\mathrm{Method}\;B}}{{\mathrm{Cor}}_{\mathrm{Method}\;A}}\;.$$

Under both ways to compute the RE, if ${\mathrm{RE}}_{\mathrm{NRMSE}}\left({\mathrm{or}\;\mathrm{RE}}_{\mathrm{Cor}}\right)\;>1,$ the best prediction performance in terms of NRMSE(or Cor) was obtained using Method B, but when ${\mathrm{RE}}_{\mathrm{NRMSE}}\left({\mathrm{or}\;\mathrm{RE}}_{\mathrm{Cor}}\right)<1,$ the best method was Method A. When ${\mathrm{RE}}_{\mathrm{NRMSE}}\;\left({\mathrm{or}\;\mathrm{RE}}_{\mathrm{Cor}}\right)\;=\;\;1,$ both methods were equally efficient.

## 3 RESULTS

Results are provided in five sections, the first three provide results for datasets EYT_2, EYT_3, and Groundnut, respectively, and the last section provides summary across all datasets. The prediction performance of using (Method B) and not using (Method A) genomic information is compared to the prediction modeling in terms of NRMSE and Pearson´s correlation (Cor).

The final section contains Supporting Information that provide results (tables and figures) for the other 11 datasets: Disease, EYT_1, Indica, Japonica, Maize, and YT_1–YT_6. General comments on the results from these datasets are given at the end of this section.

### 3.1 Dataset—EYT_2

**FIGURE 1:**
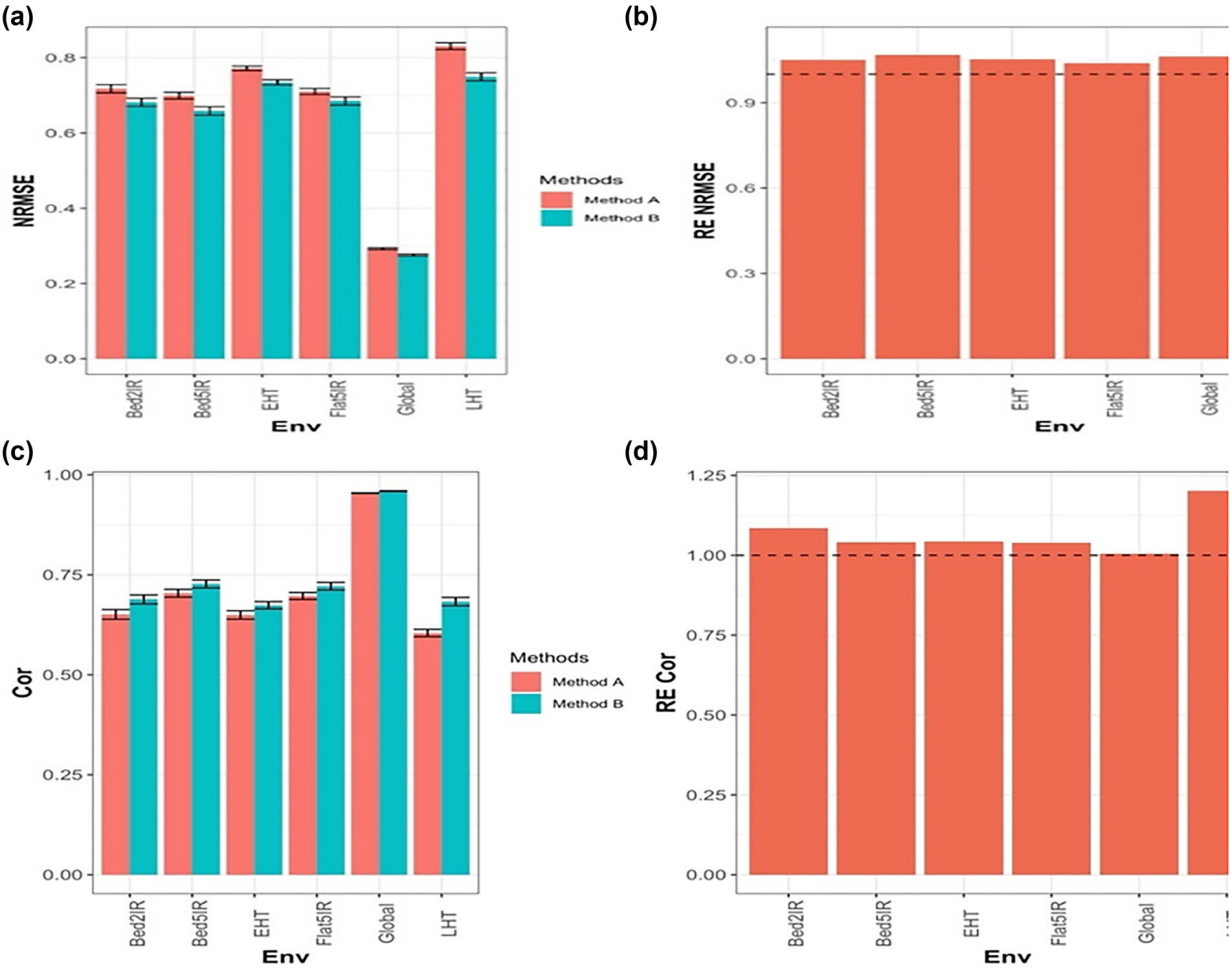
Dataset—EYT_2. (a) Prediction performance with (Method B) and without (Method A) genomic information across traits in terms of normalized root mean squared error (NRMSE) for each environment (Bed2IR, Bed5IR, EHT, Flat5IR, and LHT) and across environments (Global) under a 7‐fold random cross‐validation (7FCV) for dataset EYT_2. (b) Relative efficiency (RE) comparing Method A and Method B across traits in terms of normalized root mean squared error (NRMSE) for each environment (Bed2IR, Bed5IR, EHT, Flat5IR, and LHT) and across environments (Global) under 7FCV. (c) Prediction performance with (Method B) and without (Method A) genomic information across traits in terms of Pearson´s correlation (Cor) for each environment (Bed2IR, Bed5IR, EHT, Flat5IR, and LHT) and across environments (Global) under a 7FCV. (d) RE comparing Method A and Method B across traits in terms of Pearson´s correlation (Cor) for each environment (Bed2IR, Bed5IR, EHT, Flat5IR, and LHT) and across environments (Global) under 7FCV. Here, when RE > 1 Method B outperforms Method A in terms of prediction performance. Bed2IR, bed planting with two irrigations; Bed5IR, bed planting with five irrigations, EHT, early heat; Flat5IR, flat planting and five irrigations; LHT, late heat.

The prediction performance across traits for each environment and across environments (Global) is provided for this EYT_2′s dataset under the 7‐fold random cross‐validation (7FCV) across the four traits. Results are shown in Table 1 and Figure 1a,b in terms of NRMSE and Figures 1c,d for Cor between observed and predictive values.

**TABLE 1:** Dataset EYT_2. Prediction performance across traits with (Method B) and without (Method A) genomic information for each environment and across environments (Global) for dataset EYT_2 in terms of normalized root mean squared error (NRMSE) and Pearson correlation (Cor) under a 7FCV (7 fold cross‐validation) strategy.

Methods	Env	NRMSE	Cor	NRMSE_SE	Cor_SE	RE_NRMSE	RE_Cor
Method A	Bed2IR	0.718	0.651	0.011	0.012	–	–
Method A	Bed5IR	0.699	0.704	0.009	0.010	–	–
Method A	EHT	0.772	0.650	0.006	0.011	–	–
Method A	Flat5IR	0.710	0.697	0.008	0.009	–	–
Method A	Global	0.292	0.954	0.003	0.001	–	–
Method A	LHT	0.831	0.605	0.009	0.009	–	–
Method B	Bed2IR	0.681	0.689	0.011	0.011	1.053	1.058
Method B	Bed5IR	0.659	0.727	0.011	0.010	1.062	1.033
Method B	EHT	0.734	0.674	0.007	0.009	1.051	1.037
Method B	Flat5IR	0.685	0.722	0.011	0.010	1.037	1.035
Method B	Global	0.276	0.959	0.003	0.001	1.060	1.005
Method B	LHT	0.749	0.683	0.011	0.010	1.109	1.130

*Note*: The RE in RE_NRMSE column were computed dividing the Method A by Method B whereas the RE_Cor column were computed dividing the Method B under Method A. RE values greater than 1 indicates Method B is better in prediction performance.

Abbreviations: EHT, early heat; LHT, late heat; RE, relative efficiency; SE, standard error.

As seen in Table 1 and Figure 1a,b, Method A was worse than Method B in environments BED2IR (0.718), BED5IR (0.699), EHT (0.772), Flat5IR (0.710), LHT (0.831), and Global (0.292). Also, Method B in terms of NRMSE was better than Method A in environments BED2IR (0.681), BED5IR (0.659), EHT (0.734), Flat5IR (0.685), LHT (0.749), and Global (0.276). In terms of Cor, Method A was worse than Method B in environments BED2IR (0.651), BED5IR (0.704), EHT (0.650), Flat5IR (0.697), LHT (0.605), and Global (0.954) (Table 1 and Figure 1c,d). Method B in terms of Cor was better than Method A in environments BED2IR (0.689), BED5IR (0.727), EHT (0.674), Flat5IR (0.722), LHT (0.683), and Global (0.959).

The observed relative efficiency (RE) of comparing Method B versus Method A in terms of NRMSE for each environment and across environments were 1.053 (BED2IR), 1.062 (BED5IR), 1.051 (EHT), 1.037 (Flat5IR), 1.109 (LHT), and 1.060 (Global). This indicates Method B outperformed Method A in all environments mentioned by 5.3% (BED2IR), 6.2% (BED5IR), 5.1% (EHT), 3.7% (Flat5IR), 10.9% (LHT), and 6.0% (Global) (Table 1 and Figure 1c,d). While the RE of comparing the Method B versus Method A for each environment and across environments in terms of Cor were 1.058 (BED2IR), 1.033 (BED5IR), 1.037 (EHT), 1.035 (Flat5IR), 1.130 (LHT), and 1.005 (Global). This indicates Method B outperformed Method A in all the traits and environments in terms of Cor mentioned by 5.8% (BED2IR), 3.3% (BED5IR), 3.7% (EHT), 3.5% (Flat5IR), 13.0% (LHT), and 0.5% (Global). This indicates Method B is considerably better in prediction performance than Method A since the RE were greatly superior to one in both metrics (NRMSE and Cor).

### 3.2 Dataset—EYT_3

**FIGURE 2:**
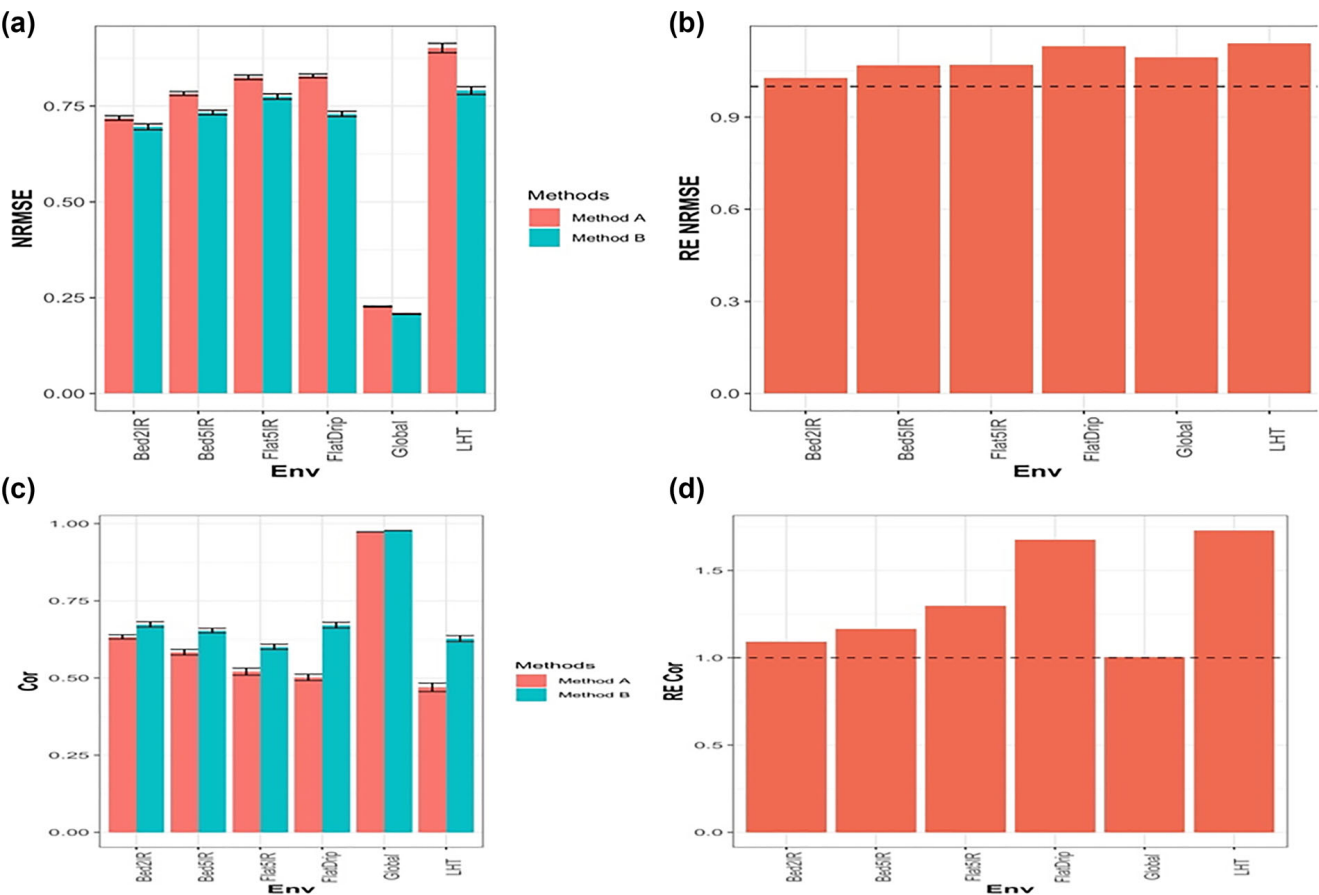
Dataset EYT_3. (a) Prediction performance with (Method B) and without (Method A) genomic information across traits in terms of normalized root mean squared error (NRMSE) for each environment (Bed2IR, Bed5IR, Flat5IR, FlatDrip, and LHT) and across environments (Global) under a 7‐fold random cross‐validation (7FCV) for dataset EYT_3. (b) Relative efficiency (RE) comparing Method A and Method B across traits in terms of normalized root mean squared error (NRMSE) for each environment (Bed2IR, Bed5IR, Flat5IR, FlatDrip, and LHT) and across environments (Global) under 7FCV. (c) Prediction performance with (Method B) and without (Method A) genomic information across traits in terms of Pearson´s correlation (Cor) for each environment (Bed2IR, Bed5IR, Flat5IR, FlatDrip, and LHT) and across environments (Global) under a 7FCV. (d) RE comparing Method A and Method B across traits in terms of Pearson´s correlation (Cor) for each environment (Bed2IR, Bed5IR, Flat5IR, FlatDrip, and LHT) and across environments (Global) under 7FCV. Here, when RE > 1 Method B outperforms Method A in terms of prediction performance. Bed2IR, bed planting with two irrigations; Bed5IR, bed planting with five irrigations, Flat5IR, flat planting and five irrigations; FlatDrip, flat planting with drip irrigation; LHT, late heat.

The prediction performance across traits for each environment and across traits and environments (Global) is provided for this EYT_3′s dataset under the 7FCV. Results are shown in Table 2 and Figure 2a,d.

**TABLE 2:** Dataset EYT_3. Prediction performance across traits with (Method B) and without (Method A) genomic information for each environment and across environments (Global) for dataset EYT_3 in terms of normalized root mean squared error (NRMSE) and Pearson correlation (Cor) under a 7FCV (7 fold cross‐validation) strategy.

Methods	Env	NRMSE	Cor	NRMSE_SE	Cor_SE	RE_NRMSE	RE_Cor
Method A	Bed2IR	0.719	0.633	0.006	0.007	–	–
Method A	Bed5IR	0.783	0.583	0.005	0.009	–	–
Method A	Flat5IR	0.825	0.521	0.006	0.012	–	–
Method A	FlatDrip	0.829	0.502	0.005	0.010	–	–
Method A	Global	0.227	0.973	0.001	0.000	–	–
Method A	LHT	0.901	0.470	0.012	0.014	–	–
Method B	Bed2IR	0.696	0.673	0.008	0.009	1.033	1.063
Method B	Bed5IR	0.733	0.653	0.006	0.008	1.068	1.120
Method B	Flat5IR	0.774	0.601	0.007	0.009	1.065	1.154
Method B	FlatDrip	0.729	0.671	0.007	0.010	1.136	1.337
Method B	Global	0.208	0.978	0.001	0.000	1.095	1.005
Method B	LHT	0.790	0.627	0.010	0.010	1.140	1.335

*Note*: The RE in RE_NRMSE column were computed dividing the Method A by Method B whereas the RE_Cor column were computed dividing the Method B under Method A. RE values greater than 1 indicates Method B is better in prediction performance.

Abbreviations: EHT, early heat; LHT, late heat; RE, relative efficiency; SE, standard error.

As seen in Table 2, in terms of NRMSE, Method A was worse in terms of prediction performance than Method B in environments BED2IR (0.719), BED5IR (0.783), Flat5IR (0.825), FlatDrip (0.829), LHT (0.901), and Global (0.227). Method B outperformed Method A in all environments BED2IR (0.696), BED5IR (0.733), Flat5IR (0.774), FlatDrip (0.729), LHT (0.790), and Global (0.208). In terms of Cor, Method A was worse than Method B in environments BED2IR (0.633), BED5IR (0.583), Flat5IR (0.521), FlatDrip (0.502), LHT (0.470), and Global (0.973). Furthermore, Method B outperformed Method A in terms of Cor in environments BED2IR (0.673), BED5IR (0.653), Flat5IR (0.601), FlatDrip (0.671), LHT (0.627), and Global (0.978). For details, see Table 2 and Figure 2a,c.

The RE of comparing Method B versus Method A under NRMSE for each environment and across environments were 1.033 (BED2IR), 1.068 (BED5IR), 1.065 (Flat5IR), 1.136 (FlatDrip), 1.140 (LHT), and 1.095 (Global). This indicates Method B outperformed Method A in all environments by 3.3% (BED2IR), 6.8% (BED5IR), 6.5% (Flat5IR), 13.6% (FlatDrip), 14.0% (LHT), and 9.5% (Global). For more details, see Table 2 and Figure 2b. While the RE of comparing Method B versus Method A for each environment and across environments in terms of Cor were 1.063 (BED2IR), 1.120 (BED5IR), 1.154 (Flat5IR), 1.337 (FlatDrip), 1.335 (LHT), and 1.005 (Global). This indicates Method B outperformed Method A in all environments in terms of Cor by 6.3% (BED2IR), 12.0% (BED5IR), 15.4% (Flat5IR), 33.7% (FlatDrip), 33.5% (LHT), and 0.5%(Global). These results indicate Method B is better in prediction performance than Method A since the RE were greatly superior to one in both metrics (NRMSE and Cor) (Table 2 and Figure 2b,d).

### 3.3 Groundnut

**FIGURE 3:**
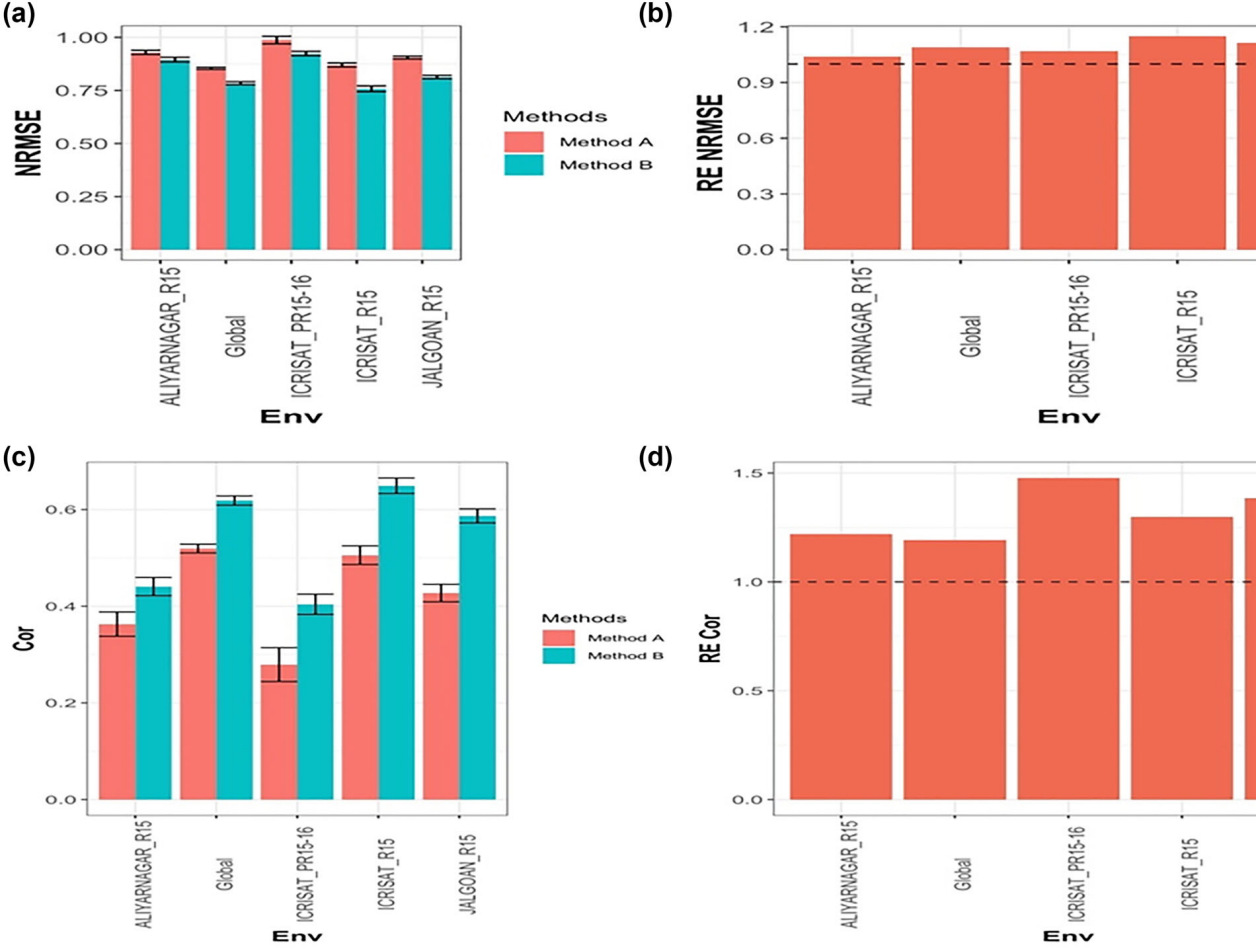
Dataset Groundnuts. (a) Prediction performance with (Method B) and without (Method A) genomic information across traits in terms of normalized root mean squared error (NRMSE) for each environment (ALIYARNAGAR_R15, ICRISAT_PR15‐16, ICRISAT_R15, and JALGOAN_R15) and across environments (Global) under a 7‐fold random cross‐validation (7FCV) for dataset Groundnut. (b) Relative efficiency (RE) comparing Method A and Method B across traits in terms of NRMSE for each environment (ALIYARNAGAR_R15, ICRISAT_PR15‐16, ICRISAT_R15, and JALGOAN_R15) and across environments (Global) under 7FCV. (c) Prediction performance with (Method B) and without (Method A) genomic information in terms of Pearson´s correlation (Cor) for each environment (ALIYARNAGAR_R15, ICRISAT_PR15‐16, ICRISAT_R15, and JALGOAN_R15) and across environments (Global) under a 7FCV. (d) RE comparing Method A and Method B in terms of Pearson´s correlation (Cor) for each environment (ALIYARNAGAR_R15, ICRISAT_PR15‐16, ICRISAT_R15, and JALGOAN_R15) and across environments (Global) under 7FCV. Here, when RE > 1 Method B outperforms Method A in terms of prediction performance.

As seen in Table 3, the NRMSE under Method A for each environment was ALIYARNAGAR_R15 (0.929), ICRISAT_PR15‐16 (0.987), ICRISAT_R15 (0.870), JALGOAN_R15 (0.905), and Global (0.854). For Method B, the environments with improved NRMSE compared to Method A were as follows: ALIYARNAGAR_R15 (0.895), ICRISAT_PR15‐16 (0.924), ICRISAT_R15 (0.758), JALGOAN_R15 (0.813), and Global (0.784). In terms of Cor, the performance of Method A in each environment was ALIYARNAGAR_R15 (0.363), ICRISAT_PR15‐16 (0.279), ICRISAT_R15 (0.506), JALGOAN_R15 (0.427), and Global (0.519). For Method B, the Cor for each environment was ALIYARNAGAR_R15 (0.441), ICRISAT_PR15‐16 (0.404), ICRISAT_R15 (0.649), JALGOAN_R15 (0.587), and Global (0.619) (Table 3 and Figure 3a,c).

**TABLE 3:** Dataset Groundnut. Prediction performance across traits with (Method B) and without (Method A) genomic information for each environment and across environments (Global) for dataset Groundnut in terms of normalized root mean squared error (NRMSE) and Pearson correlation (Cor) under a 7FCV (7 fold cross‐validation) strategy.

Methods	Env	NRMSE	Cor	NRMSE_SE	Cor_SE	RE_NRMSE	RE_Cor
Method A	ALIYARNAGAR_R15	0.929	0.363	0.010	0.025	–	–
Method A	Global	0.854	0.519	0.005	0.009	–	–
Method A	ICRISAT_PR15‐16	0.987	0.279	0.018	0.035	–	–
Method A	ICRISAT_R15	0.870	0.506	0.009	0.019	–	–
Method A	JALGOAN_R15	0.905	0.427	0.007	0.018	–	–
Method B	ALIYARNAGAR_R15	0.895	0.441	0.011	0.019	1.038	1.215
Method B	Global	0.784	0.619	0.007	0.010	1.089	1.191
Method B	ICRISAT_PR15‐16	0.924	0.404	0.010	0.021	1.069	1.447
Method B	ICRISAT_R15	0.758	0.649	0.014	0.016	1.148	1.284
Method B	JALGOAN_R15	0.813	0.587	0.008	0.015	1.113	1.373

*Note*: The RE in RE_NRMSE column were computed dividing the Method A by Method B whereas the RE_Cor column were computed dividing the Method B under Method A. RE values greater than 1 indicates Method B is better in prediction performance.

Abbreviations: RE, relative efficiency; SE, standard error.

The RE of comparing Method B versus Method A in terms of NRMSE for each environment and across environments was 1.038 (ALIYARNAGAR_R15), 1.069 (ICRISAT_PR15‐16), 1.148 (ICRISAT_R15), 1.113 (JALGOAN_R15), and 1.089 (Global). These results indicate Method B outperformed Method A in all environments by 3.8% (ALIYARNAGAR_R15), 6.9% (ICRISAT_PR15‐16), 14.8% (ICRISAT_R15), 11.3% (JALGOAN_R15), and 8.9% (Global). For more details, see Table 3 and Figure 3b. While the RE of comparing Method B versus Method A for each environment and across environments in terms of Cor was 1.215 (ALIYARNAGAR_R15), 1.447 (ICRISAT_PR15‐16), 1.284 (ICRISAT_R15), 1.373 (JALGOAN_R15), and 1.191 (Global). These results indicate Method B outperformed Method A in all environments in terms of Cor by 21.5% (ALIYARNAGAR_R15), 44.7% (ICRISAT_PR15‐16), 28.4% (ICRISAT_R15), 37.3% (JALGOAN_R15), and 19.1% (Global). Method B's prediction performance is superior to Method A since the RE's were greatly improved in both metrics (NRMSE and Cor). More details are given in Table 3 and Figure 3b,d.

### 3.4 Across datasets

**FIGURE 4:**
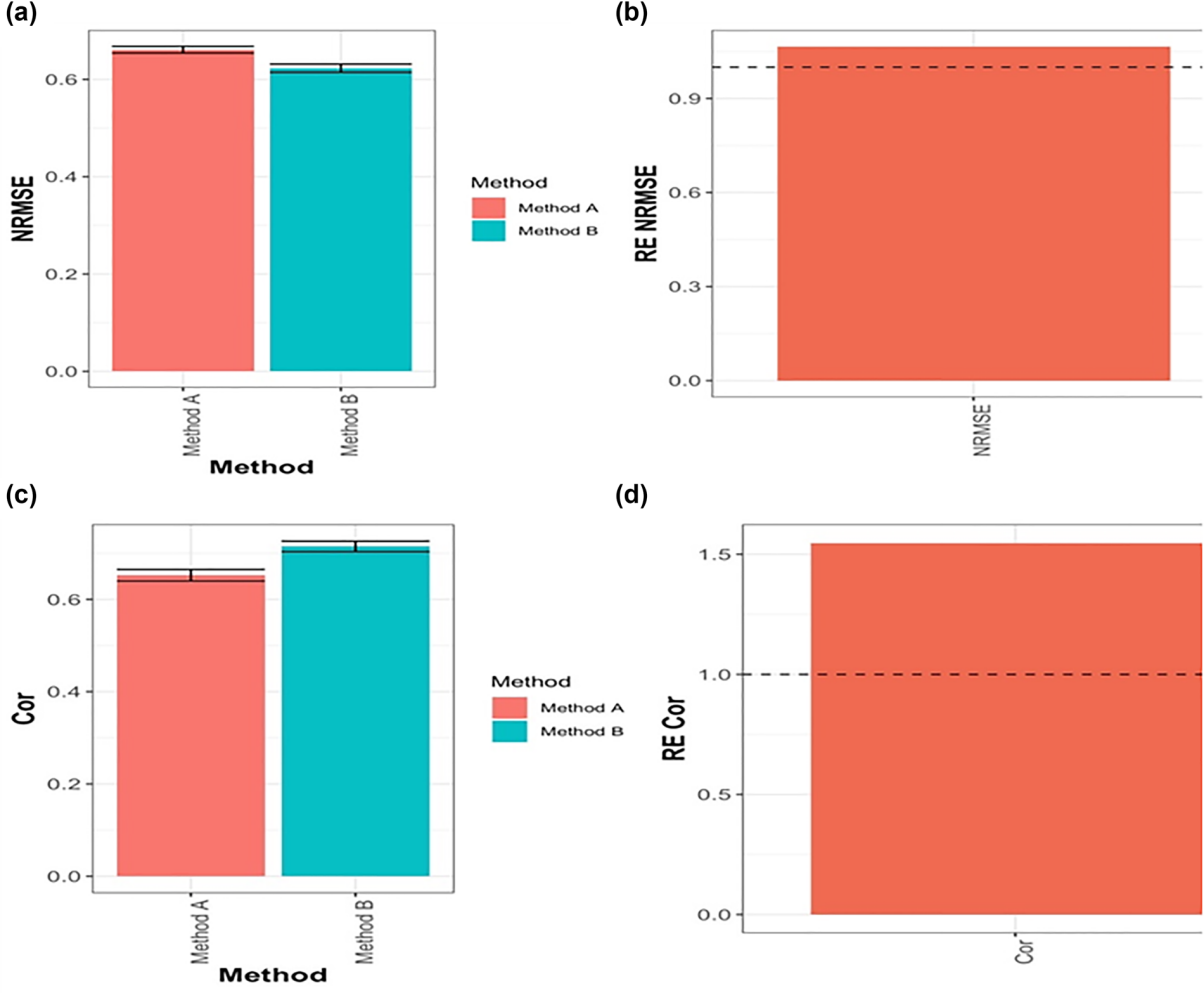
Across traits, environments, and datasets. (a) Prediction performance with (Method B) and without (Method A) genomic information across traits, environments, and datasets in terms of normalized root mean squared error (NRMSE). (b) Relative efficiency (RE) comparing Method A and Method B across traits, environments, and datasets in terms of NRMSE under a 7‐fold random cross‐validation (7FCV). (c) Prediction performance with (Method B) and without (Method A) genomic information across traits, environments, and datasets in terms of Pearson´s correlation (Cor) under a 7FCV. (d) RE comparing Method A and Method B across traits, environments, and datasets in terms of Pearson´s correlation (Cor) under 7FCV. Here, when RE > 1 Method B outperforms Method A in terms of prediction performance.

Next, we provide a meta‐analysis across traits, environments, and datasets for both metrics (NRMSE and Cor) resulting of implementing a 7FCV strategy in each dataset. As seen in Table 4, in terms of NRMSE the performance for Method A (0.832) was worse than Method B (0.781). In terms of Cor, the performance of Method A (0.170) was worse than Method B (0.248) as well. For details, see Table 4 and Figure 4a,c. The RE of comparing Method B versus Method A in terms of NRMSE strategy was 1.066, which indicates Method B outperformed Method A by 6.6% across traits, environments, and datasets. For more details, see Table 4 and Figure 4b. The RE of comparing Method B versus Method A was 1.46.1, illustrating Method B outperformed Method A by 46.1% across traits, environments, and datasets. With a higher RE in both metrics (NRMSE and Cor), Method B is clearly superior to Method A across traits, environments, and datasets (Table 4 and Figure 4d).

**TABLE 4:** Prediction performance across traits, environments and datasets with (Method B) and without (Method A) genomic information in terms of normalized root mean squared error (NRMSE) and Pearson correlation (Cor) under a 7FCV (7 fold cross‐validation) strategy.

Methods	NRMSE	Cor	NRMSE_SE	Cor_SE	RE_NRMSE	RE_Cor
Method A	0.832	0.170	0.084	0.006	–	–
Method B	0.781	0.248	0.088	0.005	1.066	1.461

*Note*: The RE in RE_NRMSE column were computed dividing the Method A by Method B whereas the RE_Cor column were computed dividing the Method B under Method A. RE values greater than 1 indicates Method B is better in prediction performance.

Abbreviations: RE, relative efficiency; SE, standard error.

### 3.5 Disease, EYT_1, Indica, Japonica, Maize, YT_1–6

Results from dataset Disease show the prediction performance across traits for each of the six locations included in the trial and based on both criteria (NRMSE and Cor) does not give Method B any superiority over Method A when genomic is not employed for the prediction of unobserved individuals (Supporting Information). Table S1 and Figure S1 show these results, specifically Figure S1B,C.

Results for wheat dataset EYT_1 show a significant advantage of the model that uses genomic information (Method B) over the non‐genomic predictions (Method A) in terms of both matrics: RE for NRMSE and Pearson's correlation between observed and predicted values. See details in Table S2 and Figure S2A–C where for each of the four environments Bed5IR, EHT, Flat5IR, LHT, and Global Genomic prediction (Method B) showed superiority over the case that genomic is not used for prediction (Method A).

Results from dataset Indica are given in in Table S3 and Figure S3A–C. Based on NRMSE, Method A was worse than Method B in years 2011, 2012, and Global but both methods gave similar prediction accuracy for predicting year 2010 (Figure S3B). However, for criterion RE_Cor Method A was worse than Method B in all 3 years (2010, 2011, and 2012) and Global. For dataset Japonica the prediction based on genomic (Method B) was superior to the method not using genomic (Method A) in 3 years 2010, 2011, and 2013 based on criterion RE NRMSE and on when predicting all the years, except 2012 based on criterion RE_Cor (Table S4, Figure S4A–D). However, for dataset Maize no increase in prediction accuracy was exhibited for Method B against Method A, as shown in Table S5 and Figure S5A–D.

For the datasets YT_1–YT_6 that included spring wheat datasets from 2013 to 2020 (Table S6–S11 and Figures S6A–D and S11A–D) the prediction given by Method B outperformed Method A for each pair of years of each of the cases, including Global. It was also observed that the clear superiority of Method B over Method A for each of these data sets is shown under both metrics used to evaluate the performance of the two methods RE_NRMSE and RE_Cor.

## 4 DISCUSSION

This study evaluated how GS prediction accuracy can increase with and without makers. For more precision, we examined this question within a multi‐environment framework (with a predictor considering environment + genotype + genotype × environment interaction) using 14 public datasets of plant breeding programs using GS.

On average, across traits, environments, and datasets within the NRMSE, we identified a genetic gain of 6.6% when using markers as opposed to their absence. For Cor, a genetic gain of 46.1% was identified when using markers, as opposed to their absence. The average of the metrics results in a genetic gain value of 26.35% when using markers as opposed to their absence.

Our results support proponents (Frouin et al., 2019; Ortiz, 2015) of GS who observe GS has the potential to transform plant and animal breeding because prediction accuracy drops an average of 26.35% when markers are not employed. The distinction between successful and unsuccessful implementation of GS can be discerned based on this variation. Several factors affect the successful implementation of the GS methodology by influencing the genomic‐enable prediction. Three of these factors are discussed as follows:

1. the relatedness of the individuals in the training and testing set (Rincent et al., 2012; Schopp et al., 2015), because unrelatedness individuals points to a low prediction accuracy, even with high‐quality markers. The degree of relatedness between individuals in the training and testing populations is important to guarantee high prediction accuracy in the context of genomic prediction because it determines the extent to which the genetic information learned from the training population can be effectively applied to the testing population. Genomic prediction models are built based on the relationship between genetic markers and the traits of interest using a training population. Therefore, if the individuals in the testing population are too closely related to those in the training population, the genomic predictions may be overly optimistic, leading to poor performance when applied to unrelated populations. On the other hand, if the individuals in the testing population are too distantly related to those in the training population, the genomic predictions may be inaccurate due to the lack of shared genetic information. Therefore, selecting an appropriate level of relatedness between the training and testing populations is essential to ensure the generalizability and accuracy of genomic prediction models (Habier et al., 2007; Rincent et al., 2012; Schopp et al., 2015);
2. the quality of markers used in genomic prediction is of paramount importance because the accuracy of genomic prediction depends on the ability of markers to capture the underlying genetic variation of the trait of interest. Inaccurate or low‐quality markers can lead to biased or imprecise predictions, resulting in reduced effectiveness and efficiency of genomic selection programs. Therefore, ensuring the quality of markers is critical in achieving the desired outcomes of genomic prediction in animal and plant breeding programs (Goddard, 2009);
3. the coverage of markers also is of paramount importance to the successful implementation of GS because it determines the extent to which the genetic variation across the genome is captured by the markers. A higher marker density and wider genome coverage result in better representation of the genetic variation, leading to more accurate and reliable predictions of an individual's genetic merit for the traits of interest. On the other hand, low marker density or uneven distribution of markers across the genome can result in biased and imprecise predictions, limiting the effectiveness of genomic selection. Therefore, ensuring adequate marker coverage is crucial for achieving the desired outcomes of genomic selection in animal and plant breeding programs (Spindel & McCouch, 2016).

The three factors just pointed out why, in terms of correlation when markers are used, an improvement of more than 46.1% is observed when using genomic compared when not using genomic. It is documented that genomic information (a) measures relatedness arising from more distant ancestors (than those included in a historical pedigree) and (b) captures the variation in realized kinship arising from the stochastic effects of Mendelian sampling and recombination (Yu et al., 2017).

Even as our results corroborate the power of GS, we are aware many breeding programs will have problems implementing GS because it depends on the ability to optimizing all potential factors. Due to the easy of measuring aditional input data such as environmental information and various data types including transcriptomics, proteomics, and metabolomics, among others, there is a resonable expectation that GS, can be implemented with significantly reduced levels of uncertainty. Increasing empirical evidence shows prediction accuracy improves significantly by adding complementary prediction model inputs. For example, Wu et al. (2022), used three types of inputs of different omics data (genomics, metabolomics, and transcriptomics,) as predictors and found the integration of the three inputs improved prediction accuracy in barley.

Furthermore, Hu et al. (2021) found that integrating three‐omics (transcriptomic, metabolomic, and genomics) data improved prediction accuracies of oat agronomic and seed nutritional traits. Also, other authors had shown integrating genomics and environmental information (Basnet et al., 2019; Costa‐Neto, Crossa et al., 2021; Costa‐Neto, Fritsche‐Neto et al., 2021; Crossa et al., 2021; Jarquin et al., 2021; Monteverde et al., 2019; Rogers & Holland, 2022; Washburn et al., 2021) improve prediction accuracy. Similarly, Atefi et al. (2021) reported the integration of phenomics and genomics enhance the prediction power of GS.

## 5 CONCLUSIONS

In this study we evaluated 14 datasets to determine the gain in prediction performance which can be achieved using genomic information in the prediction models. For each dataset we implemented the GBLUP model with the same predictor with (Method B) and without (Method A) genomic information. We found in some datasets the gain in prediction accuracy when including the genomic information was almost null, but in other datasets, the gain was more than 70%. Across traits, environments, and datasets we found that for NRMSE the gain was around 6.6% and for Cor, around 46.1%, meaning across metrics the gain is around 26.35%. Our findings corroborate that the quality, good coverage of the genomic information, and a moderate to high degree of relatedness among the individuals in the training and testing set it is of paramount importance to take advantage of GS. Our research also illustrates that the quality of genomic information and a low degree of relatedness between the training and testing set can greatly compromise the efficacy of GS.

## AUTHOR CONTRIBUTIONS

**Osval A. Montesinos‐López**: Conceptualization; investigation; analyses; writing. **Alison R. Bentley**: Investigation; methodology. **Carolina Saint Pierre**: Investigation; methodology; data curation. **Leonardo Crespo‐Herrera** Investigation; methodology; data curation; analyses. **Leonardo Rebollar‐Ruellas**; Data analyses; methodology. **Patricia Edwigis Valladares‐Celis**: Data analyses; methodology. **Morten Lillemo**: Methodology; investigation. **Abelardo Montesinos‐López**: Conceptualization; investigation; analyses; writing. **José Crossa**: Methodology; conceptualization; investigation; writing.

## CONFLICT OF INTEREST STATEMENT

The authors declare no conflicts of interest.

## Supporting information

SUPPLEMENTARY MATERIALS
