# Supplementary material for: Efficacy of plant breeding using genomic information
Source: Plant Genome. 2023 May 4;16(2):e20346. doi: 10.1002/tpg2.20346 (PMC12807367; doi:10.1002/tpg2.20346)
Supplement: Supplementary file 1 [file TPG2-16-e20346-s001.docx]

# SUPPLEMENTARY MATERIALS

| **Table S1. Dataset Disease.** Prediction performance across traits with (Method B) and without (Method A) genomic information for each environment and across environments (Global) for dataset **Disease** in terms of normalized root mean squared error (NRMSE) and Pearson Correlation (Cor) under a 7FCV (7 fold cross-validation) strategy. RE, denotes relative efficiency. The RE in RE_NRMSE (normalized root mean square error), column were computed dividing the Method A under Method B. While the RE_Cor column were computed dividing the Method B under Method A. RE values greater than one indicates Method B is better in prediction performance (SE standard error). | | | | | | | |
| --- | --- | --- | --- | --- | --- | --- | --- |
| Methods | Env | NRMSE | Cor | NRMSE_SE | Cor_SE | RE_NRMSE | RE_Cor |
| Method A | Env1 | 0.825 | 0.565 | 0.019 | 0.030 | - | - |
| Method A | Env2 | 0.828 | 0.557 | 0.023 | 0.035 | - | - |
| Method A | Env3 | 0.808 | 0.592 | 0.024 | 0.036 | - | - |
| Method A | Env4 | 0.803 | 0.592 | 0.025 | 0.031 | - | - |
| Method A | Env5 | 0.838 | 0.561 | 0.041 | 0.046 | - | - |
| Method A | Env6 | 0.815 | 0.586 | 0.021 | 0.029 | - | - |
| Method A | Global | 0.788 | 0.611 | 0.012 | 0.015 | - | - |
| ----------------------------------------------------------------------------------------------------- | | | | | | | |
| Method B | Env1 | 0.835 | 0.550 | 0.025 | 0.037 | 0.988 | 0.973 |
| Method B | Env2 | 0.837 | 0.540 | 0.023 | 0.036 | 0.988 | 0.969 |
| Method B | Env3 | 0.818 | 0.581 | 0.023 | 0.033 | 0.987 | 0.982 |
| Method B | Env4 | 0.810 | 0.584 | 0.022 | 0.029 | 0.991 | 0.987 |
| Method B | Env5 | 0.854 | 0.536 | 0.037 | 0.045 | 0.981 | 0.955 |
| Method B | Env6 | 0.827 | 0.564 | 0.018 | 0.027 | 0.985 | 0.962 |
| Method B | Global | 0.797 | 0.599 | 0.009 | 0.011 | 0.988 | 0.980 |

| **A)** | **B)** |
| --- | --- |
|  | |
| **C)** | **D)** |
|  | |

**Figure S1. Dataset Disease. A**) Prediction performance with (Method B) and without (Method A) genomic information across traits in terms of normalized root mean squared error (NRMSE) for each environment (Env1, Env2, Env3, Env4, Env5 and Env6) and across environments (Global) under a 7-fold random cross-validation (7FCV) for dataset Disease. **B**) Relative efficiency (RE) comparing Method A and B across traits in terms of normalized root mean squared error (NRMSE) for each environment (Env1, Env2, Env3, Env4, Env5 and Env6) and across environments (Global) under 7FCV. **C**) Prediction performance with (Method B) and without (Method A) genomic information across traits in terms of Pearson´s correlation (Cor) for each environment Env1, Env2, Env3, Env4, Env5 and Env6) and across environments (Global) under a 7FCV. **D**) Relative efficiency (RE) comparing Method A and B across traits in terms of Pearson´s correlation (Cor) for each environment (Env1, Env2, Env3, Env4, Env5 and Env6) and across environments (Global) under 7FCV. Where RE > 1 Method B outperforms Method A in terms of prediction performance.

| **Table S2. Dataset EYT_1.** Prediction performance across traits with (Method B) and without (Method A) genomic information across traits for each environment and across environments (Global) for dataset **EYT_1** in terms of normalized root mean squared error (NRMSE) and Pearson Correlation (Cor) under a 7FCV (7 fold cross-validation) strategy. RE, denotes relative efficiency. The RE in RE_NRMSE (normalized root mean square error), column were computed dividing the Method A under Method B. While the RE_Cor column were computed dividing the Method B under Method A. RE values greater than one indicates Method B is better in prediction performance (SE standard error). | | | | | | | |
| --- | --- | --- | --- | --- | --- | --- | --- |
| Methods | Env | NRMSE | Cor | NRMSE_SE | Cor_SE | RE_NRMSE | RE_Cor |
| Method A | Bed5IR | 0.723 | 0.681 | 0.008 | 0.012 | - | - |
| Method A | EHT | 0.803 | 0.609 | 0.005 | 0.012 | - | - |
| Method A | Flat5IR | 0.918 | 0.593 | 0.008 | 0.011 | - | - |
| Method A | Global | 0.298 | 0.952 | 0.002 | 0.001 | - | - |
| Method A | LHT | 0.824 | 0.526 | 0.012 | 0.014 | - | - |
| ------------------------------------------------------------------------------------------------------- | | | | | | | |
| Method B | Bed5IR | 0.706 | 0.685 | 0.008 | 0.009 | 1.024 | 1.006 |
| Method B | EHT | 0.763 | 0.631 | 0.007 | 0.011 | 1.052 | 1.037 |
| Method B | Flat5IR | 0.822 | 0.637 | 0.008 | 0.008 | 1.117 | 1.075 |
| Method B | Global | 0.281 | 0.958 | 0.003 | 0.001 | 1.060 | 1.006 |
| Method B | LHT | 0.739 | 0.660 | 0.011 | 0.011 | 1.115 | 1.255 |

| **A)** | **B)** |
| --- | --- |
|  | |
| **C)** | **D)** |
|  | |

**Figure S2. Dataset EYT_1. A**) Prediction performance with (Method B) and without (Method A) genomic information across traits in terms of normalized root mean squared error (NRMSE) for each environment (Bed5IR, EHT, Flat5IR and LHT) and across environments (Global) under a 7-fold random cross-validation (7FCV) strategy. Relative efficiency (RE) comparing Method A and B across traits in terms of normalized root mean squared error (NRMSE) for each environment (Bed5IR, EHT, Flat5IR and LHT) and across environments (Global). **C**) Prediction performance with (Method B) and without (Method A) genomic information across traits in terms of Pearson´s correlation (Cor) for each environment (Bed5IR, EHT, Flat5IR and LHT) and across traits and environments (Global). **D**) Relative efficiency (RE) comparing Method A and B across traits in terms of Pearson´s correlation (Cor) for each environment (Bed5IR, EHT, Flat5IR and LHT) and across environments (Global) under 7FCV. Where RE > 1 Method B outperforms Method A in terms of prediction performance.

| **Table S3. Dataset Indica.** Prediction performance across traits with (Method B) and without (Method A) genomic information for each environment and across environments and traits (Global) for dataset **Indica** in terms of normalized root mean squared error (NRMSE) and Pearson Correlation (Cor) under a 7FCV (7 fold cross-validation) strategy. RE, denotes relative efficiency. The RE in RE_NRMSE (normalized root mean square error), column were computed dividing the Method A under Method B. While the RE_Cor column were computed dividing the Method B under Method A. RE values greater than one indicates Method B is better in prediction performance (SE standard error). | | | | | | | |
| --- | --- | --- | --- | --- | --- | --- | --- |
| Methods | Env | NRMSE | Cor | NRMSE_SE | Cor_SE | RE_NRMSE | RE_Cor |
| Method A | 2010 | 0.810 | 0.500 | 0.018 | 0.026 | - | - |
| Method A | 2011 | 0.916 | 0.452 | 0.016 | 0.026 | - | - |
| Method A | 2012 | 0.874 | 0.492 | 0.011 | 0.028 | - | - |
| Method A | Global | 0.821 | 0.541 | 0.009 | 0.014 | - | - |
| ----------------------------------------------------------------------------------------------------- | | | | | | | |
| Method B | 2010 | 0.801 | 0.545 | 0.022 | 0.029 | 1.012 | 1.088 |
| Method B | 2011 | 0.878 | 0.510 | 0.019 | 0.025 | 1.043 | 1.127 |
| Method B | 2012 | 0.802 | 0.573 | 0.020 | 0.029 | 1.089 | 1.164 |
| Method B | Global | 0.773 | 0.612 | 0.013 | 0.017 | 1.063 | 1.130 |

| **A)** | **B)** |
| --- | --- |
|  | |
| **C)** | **D)** |
|  | |

**Figure S3. Dataset Indica.** Prediction performance with (Method B) and without (Method **A**) genomic information across traits in terms of normalized root mean squared error (NRMSE) for each environment (2010, 2011 and 2012) and across environments (Global) under a 7-fold random cross-validation (7FCV) for dataset **Indica**. **B**) Relative efficiency (RE) comparing Method A and B across traits in terms of normalized root mean squared error (NRMSE) for each environment (2010, 2011 and 2012) and across environments (Global) under 7FCV. **C**) Prediction performance with (Method B) and without (Method A) genomic information across traits in terms of Pearson´s correlation (Cor) for each environment (2010, 2011 and 2012) and across environments (Global) under a 7FCV. **D**) Relative efficiency (RE) comparing Method A and B across traits in terms of Pearson´s correlation (Cor) for each environment (2010, 2011 and 2012) and across environments (Global) under 7FCV. Where RE > 1 Method B outperforms Method A in terms of prediction performance.

.

| **Table S4. Dataset Japonica.** Prediction performance across traits with (Method B) and without (Method A) genomic information for each environment and across environments (Global) for dataset **Japonica** in terms of normalized root mean squared error (NRMSE) and Pearson Correlation (Cor) under a 7FCV (7 fold cross-validation) strategy. RE, denotes relative efficiency. The RE in RE_NRMSE (normalized root mean square error), column were computed dividing the Method A under Method B. While the RE_Cor column were computed dividing the Method B under Method A. RE values greater than one indicates Method B is better in prediction performance (SE standard error). | | | | | | | |
| --- | --- | --- | --- | --- | --- | --- | --- |
| Methods | Env | NRMSE | Cor | NRMSE_SE | Cor_SE | RE_NRMSE | RE_Cor |
| Method A | 2009 | 1.000 | 0.448 | 0.052 | 0.055 | - | - |
| Method A | 2010 | 0.918 | 0.557 | 0.021 | 0.019 | - | - |
| Method A | 2011 | 0.820 | 0.596 | 0.017 | 0.024 | - | - |
| Method A | 2012 | 0.835 | 0.536 | 0.021 | 0.030 | - | - |
| Method A | 2013 | 0.805 | 0.652 | 0.041 | 0.038 | - | - |
| Method A | Global | 0.472 | 0.880 | 0.010 | 0.005 | - | - |
| ------------------------------------------------------------------------------------------------------- | | | | | | | |
| Method B | 2009 | 1.017 | 0.435 | 0.056 | 0.054 | 0.983 | 0.972 |
| Method B | 2010 | 0.857 | 0.597 | 0.023 | 0.023 | 1.071 | 1.072 |
| Method B | 2011 | 0.777 | 0.624 | 0.017 | 0.021 | 1.055 | 1.047 |
| Method B | 2012 | 0.839 | 0.518 | 0.021 | 0.027 | 0.994 | 0.966 |
| Method B | 2013 | 0.754 | 0.683 | 0.039 | 0.036 | 1.068 | 1.048 |
| Method B | Global | 0.462 | 0.885 | 0.010 | 0.006 | 1.022 | 1.005 |

| **A)** | **B)** |
| --- | --- |
|  | |
| **C)** | **D)** |
|  | |

**Figure S4. Dataset Japonica. A**) Prediction performance with (Method B) and without (Method A) genomic information across traits in terms of normalized root mean squared error (NRMSE) for each environment (2010, 2011, 2012 and 2013) and across environments (Global) under a 7-fold random cross-validation (7FCV) for data dataset **Japonica**. **B**) Relative efficiency (RE) comparing Method A and B across traits in terms of normalized root mean squared error (NRMSE) for each environment (2010, 2011, 2012 and 2013) and across environments (Global) under 7FCV. **C)** Prediction performance with (Method B) and without (Method A) genomic information across traits in terms of Pearson´s correlation (Cor) for each environment (2010, 2011, 2012 and 2013) and across environments (Global) under a 7FCV. **D**) Relative efficiency (RE) comparing Method A and B across traits in terms of Pearson´s correlation (Cor) for each environment (2010, 2011, 2012 and 2013) and across environments (Global) under 7FCV. Where RE > 1 Method B outperforms Method A in terms of prediction performance.

| **Table S5. Dataset Maize.** Prediction performance across traits with (Method B) and without (Method A) genomic information for each environment and across environments (Global) for dataset **Maize** in terms of normalized root mean squared error (NRMSE) and Pearson Correlation (Cor) under a 7FCV (7 fold cross-validation) strategy. RE, denotes relative efficiency. The RE in RE_NRMSE (normalized root mean square error), column were computed dividing the Method A under Method B. While the RE_Cor column were computed dividing the Method B under Method A. RE values greater than one indicates Method B is better in prediction performance (SE standard error). | | | | | | | |
| --- | --- | --- | --- | --- | --- | --- | --- |
| Methods | Env | NRMSE | Cor | NRMSE_SE | Cor_SE | RE_NRMSE | RE_Cor |
| Method A | 1 | 0.949 | 0.527 | 0.028 | 0.028 | - | - |
| Method A | 2 | 0.855 | 0.536 | 0.008 | 0.015 | - | - |
| Method A | 3 | 0.908 | 0.503 | 0.026 | 0.031 | - | - |
| Method A | 4 | 0.880 | 0.557 | 0.010 | 0.036 | - | - |
| Method A | Global | 0.865 | 0.503 | 0.007 | 0.014 | - | - |
| ------------------------------------------------------------------------------------------------------ | | | | | | | |
| Method B | 1 | 0.960 | 0.323 | 0.005 | 0.016 | 0.988 | 0.613 |
| Method B | 2 | 0.930 | 0.365 | 0.012 | 0.031 | 0.919 | 0.681 |
| Method B | 3 | 0.945 | 0.365 | 0.015 | 0.038 | 0.961 | 0.725 |
| Method B | 4 | 0.897 | 0.446 | 0.008 | 0.022 | 0.980 | 0.801 |
| Method B | Global | 0.914 | 0.408 | 0.007 | 0.017 | 0.947 | 0.811 |

| **A)** | **B)** |
| --- | --- |
|  | |
| **C)** | **D)** |
|  | |

**Figure S5. Dataset Maize. A**) Prediction performance with (Method B) and without (Method A) genomic information in terms of normalized root mean squared error (NRMSE) for each environment (1, 2, 3 and 4) and across environments (Global) under a 7-fold random cross-validation (7FCV) for dataset **Maize**. **B**) Relative efficiency (RE) comparing Method A and B in terms of normalized root mean squared error (NRMSE) for each environment (1, 2, 3 and 4) and across environments (Global) under 7FCV. **C**) Prediction performance with (Method B) and without (Method A) genomic information in terms of Pearson´s correlation (Cor) for each environment (1, 2, 3 and 4) and across environments (Global) under a 7FCV. **D**) Relative efficiency (RE) comparing Method A and B in terms of Pearson´s correlation (Cor) for each environment (1, 2, 3 and 4) and across environments (Global) under 7FCV. Where RE > 1 Method B outperforms Method A in terms of prediction performance.

| **Table S6. Dataset** **YT_1.** Prediction performance with (Method B) and without (Method A) genomic information for each environment and across environments (Global) for dataset **YT_1** in terms of normalized root mean squared error (NRMSE) and Pearson Correlation (Cor) under a 7FCV (7 fold cross-validation) strategy. RE, denotes relative efficiency. The RE in RE_NRMSE (normalized root mean square error), column were computed dividing the Method A under Method B. While the RE_Cor column were computed dividing the Method B under Method A. RE values greater than one indicates Method B is better in prediction performance (SE standard error). | | | | | | | |
| --- | --- | --- | --- | --- | --- | --- | --- |
| Methods | Env | NRMSE | NRMSE_SE | Cor | Cor_SE | RE_NRMSE | RE_Cor |
| Method A | YT_14_15 | 0.892 | 0.012 | 0.472 | 0.024 | - | - |
| Method A | YT_13_14 | 0.957 | 0.008 | 0.290 | 0.032 | - | - |
| Method A | Global | 0.519 | 0.009 | 0.855 | 0.005 | - | - |
| ---------------------------------------------------------------------------------------------------------- | | | | | | | |
| Method B | YT_14_15 | 0.811 | 0.012 | 0.590 | 0.017 | 1.100 | 1.250 |
| Method B | YT_13_14 | 0.871 | 0.012 | 0.499 | 0.017 | 1.099 | 1.721 |
| Method B | Global | 0.472 | 0.008 | 0.882 | 0.004 | 1.100 | 1.032 |

| **A)** | **B)** |
| --- | --- |
|  | |
| **C)** | **D)** |
|  | |

**Figure S6. Dataset YT_1**. **A**) Prediction performance with (Method B) and without (Method A) genomic information across traits in terms of normalized root mean squared error (NRMSE) for each environment (YT_13_14 and YT_14_15) and across environments (Global) under a 7-fold random cross-validation (7FCV) for dataset **YT_1**. **B**) Relative efficiency (RE) comparing Method A and B across traits in terms of normalized root mean squared error (NRMSE) for each environment (YT_13_14 and YT_14_15) and across environments (Global) under 7-fold random cross-validation (7FCV). **C**) Prediction performance with (Method B) and without (Method A) genomic information across traits in terms of Pearson´s correlation (Cor) for each environment (YT_13_14 and YT_14_15) and across environments (Global) under a 7FCV. **D**) Relative efficiency (RE) comparing Method A and B across traits in terms of Pearson´s correlation (Cor) for each environment (YT_13_14 and YT_14_15) and across environments (Global) under 7FCV. Where RE > 1 Method B outperforms Method A in terms of prediction performance.

| **Table S7. Dataset YT_2.** Prediction performance with (Method B) and without (Method A) genomic information for each environment and across environments (Global) for dataset **YT_2** in terms of normalized root mean squared error (NRMSE) and Pearson Correlation (Cor) under a 7FCV (7 fold cross-validation) strategy. RE, denotes relative efficiency. The RE in RE_NRMSE (normalized root mean square error), column were computed dividing the Method A under Method B. While the RE_Cor column were computed dividing the Method B under Method A. RE values greater than one indicates Method B is better in prediction performance (SE standard error). | | | | | | | |
| --- | --- | --- | --- | --- | --- | --- | --- |
| Methods | Env | NRMSE | NRMSE_SE | Cor | Cor_SE | RE_NRMSE | RE_Cor |
| Method A | YT_15_16 | 0.979 | 0.006 | 0.216 | 0.035 | - | - |
| Method A | YT_14_15 | 0.991 | 0.008 | 0.154 | 0.031 | - | - |
| Method A | Global | 0.635 | 0.008 | 0.774 | 0.007 | - | - |
| ---------------------------------------------------------------------------------------------------------- | | | | | | | |
| Method B | YT_15_16 | 0.892 | 0.013 | 0.454 | 0.034 | 1.097 | 2.098 |
| Method B | YT_14_15 | 0.949 | 0.016 | 0.329 | 0.033 | 1.044 | 2.134 |
| Method B | Global | 0.596 | 0.007 | 0.803 | 0.005 | 1.065 | 1.039 |

| **A)** | **B)** |
| --- | --- |
|  | |
| **C)** | **D)** |
|  | |

**Figure S7. Dataset YT_2. A**) Prediction performance with (Method B) and without (Method A) genomic information across traits in terms of normalized root mean squared error (NRMSE) for each environment (YT_14_15 and YT_15_16) and across environments (Global) under a 7-fold random cross-validation (7FCV) for data dataset yt_3Wheat_2). **B**) Relative efficiency (RE) comparing Method A and B across traits in terms of normalized root mean squared error (NRMSE) for each environment (YT_14_15 and YT_15_16) and across environments (Global) under 7FCV. **C**) Prediction performance with (Method B) and without (Method A) genomic information across traits in terms of Pearson´s correlation (Cor) for each environment (YT_14_15 and YT_15_16) and across environments (Global) under a 7FCV. **D**) Relative efficiency (RE) comparing Method A and B across traits in terms of Pearson´s correlation (Cor) for each environment (YT_14_15 and YT_15_16) and across environments (Global) under 7FCV. Where RE > 1 Method B outperforms Method A in terms of prediction performance.

| **Table S8. Dataset YT_3.** Prediction performance with (Method B) and without (Method A) genomic information for each environment and across environments (Global) for dataset **YT_3** in terms of normalized root mean squared error (NRMSE) and Pearson Correlation (Cor) under a 7FCV (7 fold cross-validation) strategy. RE, denotes relative efficiency. The RE in RE_NRMSE (normalized root mean square error), column were computed dividing the Method A under Method B. While the RE_Cor column were computed dividing the Method B under Method A. RE values greater than one indicates Method B is better in prediction performance (SE standard error). | | | | | | | |
| --- | --- | --- | --- | --- | --- | --- | --- |
| Methods | Env | NRMSE | NRMSE_SE | Cor | Cor_SE | RE_NRMSE | RE_Cor |
| Method A | YT_16_17 | 0.980 | 0.005 | 0.226 | 0.053 | - | - |
| Method A | YT_15_16 | 0.989 | 0.006 | 0.164 | 0.029 | - | - |
| Method A | Global | 0.599 | 0.011 | 0.801 | 0.008 | - | - |
| --------------------------------------------------------------------------------------------------------- | | | | | | | |
| Method B | YT_16_17 | 0.883 | 0.012 | 0.474 | 0.023 | 1.110 | 2.092 |
| Method B | YT_15_16 | 0.858 | 0.009 | 0.516 | 0.015 | 1.153 | 3.143 |
| Method B | Global | 0.531 | 0.012 | 0.848 | 0.008 | 1.126 | 1.058 |

| **A)** | **B)** |
| --- | --- |
|  | |
| **C)** | **D)** |
|  | |

**Figure S8. Data set YT_3. A**) Prediction performance with (Method B) and without (Method A) genomic information across traits in terms of normalized root mean squared error (NRMSE) for each environment (YT_15_16 and YT_16_17) and across environments (Global) under a 7-fold random cross-validation (7FCV) for data dataset 11 (Wheat_3). **B**) Relative efficiency (RE) comparing Method A and B across traits in terms of normalized root mean squared error (NRMSE) for each environment (YT_15_16 and YT_16_17) and across environments (Global) under 7FCV. **C**) Prediction performance with (Method B) and without (Method A) genomic information across traits in terms of Pearson´s correlation (Cor) for each environment (YT_15_16 and YT_16_17) and across environments (Global) under a 7FCV. **D**) Relative efficiency (RE) comparing Method A and B across traits in terms of Pearson´s correlation (Cor) for each environment (YT_15_16 and YT_16_17) and across environments (Global) under 7FCV. Where RE > 1 Method B outperforms Method A in terms of prediction performance.

| **Table S9. Data set YT_4.** Prediction performance with (Method B) and without (Method A) genomic information for each environment and across environments (Global) for dataset 12 (Wheat_4) in terms of normalized root mean squared error (NRMSE) and Pearson Correlation (Cor) under a 7FCV (7 fold cross-validation) strategy. RE, denotes relative efficiency. The RE in RE_NRMSE (normalized root mean square error), column were computed dividing the Method A under Method B. While the RE_Cor column were computed dividing the Method B under Method A. RE values greater than one indicates Method B is better in prediction performance (SE standard error). | | | | | | | |
| --- | --- | --- | --- | --- | --- | --- | --- |
| Methods | Env | NRMSE | NRMSE_SE | Cor | Cor_SE | RE_NRMSE | RE_Cor |
| Method A | YT_17_18 | 0.967 | 0.006 | 0.272 | 0.018 | - | - |
| Method A | YT_16_17 | 0.985 | 0.004 | 0.184 | 0.022 | - | - |
| Method A | Global | 0.961 | 0.006 | 0.282 | 0.022 | - | - |
| ---------------------------------------------------------------------------------------------------------- | | | | | | | |
| Method B | YT_17_18 | 0.900 | 0.015 | 0.447 | 0.037 | 1.075 | 1.644 |
| Method B | YT_16_17 | 0.916 | 0.009 | 0.411 | 0.018 | 1.076 | 2.227 |
| Method B | Global | 0.896 | 0.010 | 0.449 | 0.020 | 1.073 | 1.593 |

| **A)** | **B)** |
| --- | --- |
|  | |
| **C)** | **D)** |
|  | |

**Figure S9. Dataset YT_4. A**) Prediction performance with (Method B) and without (Method A) genomic information across traits in terms of normalized root mean squared error (NRMSE) for each environment (YT_16_17 and YT_17_18) and across environments (Global) under a 7-fold random cross-validation (7FCV) for data dataset **YT_4**. **B**) Relative efficiency (RE) comparing Method A and B across traits in terms of normalized root mean squared error (NRMSE) for each environment (YT_16_17 and YT_17_18) and across environments (Global) under 7FCV. **C**) Prediction performance with (Method B) and without (Method A) genomic information across traits in terms of Pearson´s correlation (Cor) for each environment (YT_16_17 and YT_17_18) and across environments (Global) under a 7FCV. **D**) Relative efficiency (RE) comparing Method A and B across traits in terms of Pearson´s correlation (Cor) for each environment (YT_16_17 and YT_17_18) and across environments (Global) under 7FCV. Where RE > 1 Method B outperforms Method A in terms of prediction performance.

.

| **Table S10. Dataset YT_5.** Prediction performance with (Method B) and without (Method A) genomic information for each environment and across environments (Global) for dataset **YT_5** in terms of normalized root mean squared error (NRMSE) and Pearson Correlation (Cor) under a 7FCV (7 fold cross-validation) strategy. RE, denotes relative efficiency. The RE in RE_NRMSE (normalized root mean square error), column were computed dividing the Method A under Method B. While the RE_Cor column were computed dividing the Method B under Method A. RE values greater than one indicates Method B is better in prediction performance (SE standard error). | | | | | | | |
| --- | --- | --- | --- | --- | --- | --- | --- |
| Methods | Env | NRMSE | NRMSE_SE | Cor | Cor_SE | RE_NRMSE | RE_Cor |
| Method A | YT_18_19 | 0.929 | 0.009 | 0.412 | 0.041 | - | - |
| Method A | YT_17_18 | 0.964 | 0.013 | 0.274 | 0.040 | - | - |
| Method A | Global | 0.932 | 0.007 | 0.373 | 0.021 | - | - |
| ---------------------------------------------------------------------------------------------------------------- | | | | | | | |
| Method B | YT_18_19 | 0.859 | 0.014 | 0.517 | 0.029 | 1.082 | 1.256 |
| Method B | YT_17_18 | 0.879 | 0.016 | 0.486 | 0.022 | 1.096 | 1.773 |
| Method B | Global | 0.861 | 0.011 | 0.511 | 0.018 | 1.082 | 1.371 |

| **A)** | **B)** |
| --- | --- |
|  | |
| **C)** | **D)** |
|  | |

**Figure S10. Dataset YT_5. A**) Prediction performance with (Method B) and without (Method A) genomic information across traits in terms of normalized root mean squared error (NRMSE) for each environment (YT_17_18 and YT_18_19) and across environments (Global) under a 7-fold random cross-validation (7FCV) for data dataset 13 (Wheat_5). **B**) Relative efficiency (RE) comparing Method A and B across traits in terms of normalized root mean squared error (NRMSE) for each environment (YT_17_18 and YT_18_19) and across environments (Global) under 7FCV. **C**) Prediction performance with (Method B) and without (Method A) genomic information across traits in terms of Pearson´s correlation (Cor) for each environment (YT_17_18 and YT_18_19) and across environments (Global) under a 7FCV. **D**) Relative efficiency (RE) comparing Method A and B across traits in terms of Pearson´s correlation (Cor) for each environment (YT_17_18 and YT_18_19) and across environments (Global) under 7FCV. Where RE > 1 Method B outperforms Method A in terms of prediction performance.

| **Table S11. Dataset YT_6.** Prediction performance with (Method B) and without (Method A) genomic information for each environment and across environments (Global) for dataset 14 (Wheat_6) in terms of normalized root mean squared error (NRMSE) and Pearson Correlation (Cor) under a 7FCV (7 fold cross-validation) strategy. RE, denotes relative efficiency. The RE in RE_NRMSE (normalized root mean square error), column were computed dividing the Method A under Method B. While the RE_Cor column were computed dividing the Method B under Method A. RE values greater than one indicates Method B is better in prediction performance (SE standard error). | | | | | | | |
| --- | --- | --- | --- | --- | --- | --- | --- |
| Methods | Env | NRMSE | NRMSE_SE | Cor | Cor_SE | RE_NRMSE | RE_Cor |
| Method A | YT_19_20 | 0.987 | 0.027 | 0.179 | 0.100 | - | - |
| Method A | YT_18_19 | 0.999 | 0.003 | 0.043 | 0.028 | - | - |
| Method A | Global | 0.558 | 0.012 | 0.830 | 0.008 | - | - |
| -------------------------------------------------------------------------------------------------------------- | | | | | | | |
| Method B | YT_19_20 | 0.901 | 0.027 | 0.459 | 0.071 | 1.096 | 2.559 |
| Method B | YT_18_19 | 0.846 | 0.017 | 0.533 | 0.029 | 1.182 | 12.281 |
| Method B | Global | 0.475 | 0.016 | 0.880 | 0.009 | 1.175 | 1.060 |

| **A)** | **B)** |
| --- | --- |
|  | |
| **C)** | **D)** |
|  | |

**Figure S11. Dataset YT_6. A**) Prediction performance with (Method B) and without (Method A) genomic information across traits in terms of normalized root mean squared error (NRMSE) for each environment (YT_18_19 and YT_19_20) and across environments (Global) under a 7-fold random cross-validation (7FCV) for data dataset 14 (Wheat_6). **B**) Relative efficiency (RE) comparing Method A and B across traits in terms of normalized root mean squared error (NRMSE) for each environment (YT_18_19 and YT_19_20) and across environments (Global) under 7FCV. **C**) Prediction performance with (Method B) and without (Method A) genomic information across traits in terms of Pearson´s correlation (Cor) for each environment (YT_18_19 and YT_19_20) and across environments (Global) under a 7FCV. **D**) Relative efficiency (RE) comparing Method A and B across traits in terms of Pearson´s correlation (Cor) for each environment (YT_18_19 and YT_19_20) and across environments (Global) under 7FCV. Where RE > 1 Method B outperforms Method A in terms of prediction performance.
